# New Indices for Predicting Glycaemic Variability

**DOI:** 10.1371/journal.pone.0046517

**Published:** 2012-09-27

**Authors:** Akifumi Ogawa, Akinori Hayashi, Eriko Kishihara, Sonomi Yoshino, Akihiro Takeuchi, Masayoshi Shichiri

**Affiliations:** 1 Department of Endocrinology, Diabetes and Metabolism, School of Medicine, Kitasato University, Kanagawa, Japan; 2 Department of Medical Informatics, School of Allied Health Sciences, Kitasato University, Kanagawa, Japan; University of Michigan Medical School, United States of America

## Abstract

Blood glucose variability is known to be associated with increased risk of long-term complications. Reliable indices for predicting hyperglycaemic and hypoglycaemic fluctuations are therefore needed. Glycaemic standard deviation (SD) obtained by continuous glucose monitoring correlates closely with nine previously described glycaemic variability formulas. Here, new indices predictive of glycaemic variability were developed, which can be calculated from laboratory measures based on a single blood draw. The indices included the glycated albumin (GA) to HbA1c ratio (GA/A1c ratio) and the fasting C-peptide immunoreactivity (FCPR) to fasting plasma glucose (FPG) ratio (FCPR index). Predictive values of these indices were assessed in 100 adults with diabetes. GA/A1c ratio and FCPR index showed close associations with glycaemic SD in addition to the nine existing glucose variability formulas. Subjects with a GA/A1c ratio ≥2.8 and FCPR index <3.0 showed the greatest SD and longest durations of hypoglycaemia, while those with a GA/A1c ratio <2.8 and FCPR index ≥3.0 had smaller SDs and little sign of hypoglycaemia. In adults with diabetes, a high GA/A1c ratio and low FCPR index value reflect higher glycaemic excursions, irrespective of diabetes type. Simultaneous measurements of GA, HbA1c, FPG and FCPR may help to identify a group of patients who warrant closer monitoring in relation to glycaemic variability and hypoglycaemia.

## Introduction

The predominant focus of diabetic therapy has long been on lowering HbA1c (A1c) levels, with a strong emphasis on fasting plasma glucose (FPG), but the role of postprandial hyperglycaemia and glucose variability in relation to the risk of cardiovascular disease has recently been the subject of intense debate [1], [2]. Glycaemic variability influences endothelial function in both non-diabetic and type 2 diabetic subjects [3], accounting for increased cardiovascular risk [4]. Because A1c reflects the average blood glucose level over the preceding 1–2 month period [5], unexpectedly high or low blood glucose levels and blood glucose variability are not detected [6], [7]. Since continuous glucose monitoring (CGM) is not yet available for daily use in most patients, reliable indices predictive of marked glycaemic fluctuations would help to identify patients who may benefit from closer monitoring and modified therapeutic manoeuvres.

Glycated albumin (GA) levels are expected to reflect average blood glucose level over the previous 1–2 weeks, given that the half-life of albumin is approximately 17 days [8]. In addition, because GA increases as the blood glucose level is elevated [9] and the rate of glycation is 10 times faster than that of haemoglobin [10], serum GA levels may also be affected by temporarily high blood glucose spikes. A previous study using CGM suggested an association between GA, but not A1c, and blood glucose variability [11]. The GA/A1c ratio increases when basal β-cell function is reduced [12], while glycaemic variability is reported to correlate with β-cell dysfunction [13]. Further, GA/A1c ratio is associated with fasting C-peptide immunoreactivity (FCPR) to FPG ratio (FCPR index) in type 2 diabetic patients [14], [15]. Based on these results, we hypothesized that basal β-cell function and GA/A1c ratio could serve as clinical indices to predict blood glucose variability. We thus introduce the use of FCPR index combined with GA/A1c ratio as potential predictors of glycaemic variability.

## Patients and Methods

From a total of approximately 600 type 1 and type 2 diabetic patients followed by the authors at the outpatient clinic of the Kitasato University Hospital, those showing either a previous history of hypoglycaemia or any record of postprandial hyperglycaemia on self-monitoring of blood glucose were asked to participate in this trial. A total of 203 patients agreed to perform 72-h CGM using the CGMS system GOLD (Medtronic Minimed Inc. Northridge, CA) and to perform blood tests on the last morning of CGM. These included a complete blood count, serum biochemical analysis for more than 15 items, anti-glutamic acid decarboxylase antibody, GA, A1c, FPG, FCPR, and fasting serum insulin. All blood test values were examined to identify any relationship with glycaemic variability. Only GA, A1c, FPG, and FCPR revealed any significant association with glycaemic standard deviation (SD). This initial analysis enrolling 193 patients, excluding 10 patients who failed to obtain accurate CGM recording, indicated that glycaemic SD was closely correlated with both GA/A1c ratio (r = 0.39, *P*<0.0001) and FCPR index (r = −0.39, *P*<0.0001). GA/A1c ratio and FCPR index were identified as potentially powerful indices predicting glycaemic variability, and these were reanalyzed after carefully excluding patients with altered albumin and/or haemoglobin metabolism that could affect A1c and GA values. These included patients with conditions such as malignancy, liver cirrhosis, haematological diseases (including those being treated), persistent proteinuria, impaired renal function, thyroid dysfunction, secondary diabetes and pregnancy. Patients showing acute metabolic complications of diabetes and fulminant type 1 diabetes were also excluded. Full 72-h CGM recordings and a blood sample as described below were obtained from 100 patients. All patients provided written consent to allow their samples and CGM recordings to be analyzed, and all patients remained anonymous. The protocol was approved by the Kitasato University Medical Ethics Committee. There were 44 patients with type 1 and 56 with type 2 diabetes, including 39 males and 61 females with a mean age of 53.0±17.1 years. Their average FPG, A1c, GA and FCPR values were 10.0±4.3 mmol/l, 73±20 mmol/mol (8.9±1.8%, National Glycohemoglobin Standardization Program (NGSP) equivalent values), 25.9±6.4% and 0.3±0.3 nmol/l, respectively (Table 1). The median FCPR value of the 100 diabetic patients was 0.13 nmol/l (range 0.001–1.40 nmol/l). All 44 type 1 patients were treated with insulin and this group contained more patients with very low FCPR values (median 0.01 nmol/l, range 0.001–0.20 nmol/l), while 38 of 56 type 2 patients with a median FCPR value of 0.47 nmol/l (range 0.001–1.40 nmol/l) were on insulin. Of the 72-h CGM data, recordings for the 48 h starting from the next 12:00 a.m. after wearing the CGM device were used.

**Table 1:** Characteristics of 100 diabetic patients.

	All	Type 1	Type 2	*P* value
Number	100	44	56	
Sex (M/F)	39/61	13/31	26/30	0.0838*
Age (years)	53.0±17.1	49.5±17.2	55.9±16.7	0.0631¶
Height (cm)	160.0±9.7	160.1±9.8	160.0±9.7	0.9438¶
Weight (kg)	59.2±14.7	55.7±10.7	62.1±16.8	0.0289¶
BMI (kg/m^2^)	23.0±4.2	21.7±3.4	24.0±4.5	0.0046¶
Duration of diabetes (years)	13.0±8.8	12.2±8.5	13.7±9.2	0.4018¶
FPG (mmol/l)	10.0±4.3	10.9±4.7	9.3±3.7	0.0496¶
FCPR (nmol/l)	0.3±0.3	0.03±0.05	0.5±0.3	<0.0001¶
HbA_1c_ (mmol/mol, IFCC)	73±20	72±20	75±20	0.4104¶
HbA_1c_ (%, NGSP)	8.9±1.8	8.7±1.8	9.0±1.8	0.4245¶
GA (%)	25.9±6.4	27.5±6.2	24.6±6.4	0.0279¶
Average glucose value (mmol/l)	9.5±2.9	9.8±2.8	9.2±2.9	0.3424¶
Glycaemic SD (mmol/l)	3.0±1.2	3.6±1.0	2.5±1.0	<0.0001¶
GA/HbA_1c_ ratio	2.9±0.5	3.2±0.4	2.7±0.5	<0.0001¶
FCPR index	3.4±4.2	0.4±0.7	5.6±4.5	<0.0001¶
Treatment				
Insulin	82	44	38	
Oral reagents only	18	0	18	

Data are mean ± SD. *P* value: type 1 diabetes *vs.* type 2 diabetes,

* χ^2^ test,

¶ unpaired t-test.

BMI, body mass index; GA, glycated albumin; FPG, fasting plasma glucose; FCPR, fasting C-peptide immunoreactivity; Glycaemic SD, glycaemic standard deviation; GA/A1c ratio, GA to HbA_1c_ ratio; FCPR index, FCPR to FPG ratio (FCPR/FPG×100).

Glycaemic SD was calculated using all 576 glucose values during 48-h CGM recordings obtained at 5-min intervals. The following formulas described in the literature were determined, in addition to SD values: % coefficient of variation (%CV) [16], range (difference between the highest and lowest glucose levels) [16], interquartile range (75th–25th percentile) [16], J-index (combination of information from mean and SD of all glucose values) [17], mean amplitude of glycaemic excursions (average amplitude of upstrokes or downstrokes with magnitude greater than 1 SD) [18], M_R_ (dissociation degree from the ideal blood glucose level [default value of ideal blood glucose “R” = 5.6 mmol/l]) [19], index of glycaemic control (sum of hyperglycaemic index and hypoglycaemic index, an indicator of the deviation between the measured glucose values and the set threshold values for hyperglycaemia (default value of 7.8 mmol/l) and hypoglycaemia (default value of 4.4 mmol/l)) [16], mean of daily difference (mean difference between glucose values at the same time of day on 2 consecutive days under standardized conditions) [20], and continuous overlapping net glycaemic action (CONGA_n_, the SD of the difference between values obtained exactly n hours apart; CONGA_1–24_ is the average of CONGA_n_ for all integers of n from 1–24) [21]. These indices were semi-automatically calculated from CGM data using Excel with a customized module of visual basic application. A1c was measured as Japan Diabetes Society values by high-performance liquid chromatography using an automated system HLC-723G8 (Tosoh Co., Tokyo, Japan, %CV of intra-assay variability <0.3%), and equivalent values were expressed as both International Federation of Clinical Chemistry and Laboratory Medicine (IFCC) units (mmol/mol) [22] and NGSP values (%) [23]. FPG, GA and FCPR were measured by the glucose oxidase peroxidase method, improved bromocresol purple method, and chemiluminescent enzyme immunoassay method using an automated system GA08II (A&T Co., Kanagawa, Japan, %CV <0.8%), Lucica™ glycated albumin-L assay kit (Asahi Kasei Pharma, Japan, %CV <3%) and Lumipulse Presto® C-peptide commercial kit (Fujirebio Co., Tokyo, Japan, %CV <10%), respectively.

We used GraphPad Prism 5.02 software (GraphPad Software Inc. San Diego, CA) for statistical analysis. Univariate analyses were performed to identify correlations between 48-h glycaemic SD data and patient characteristics or diabetic indices calculated from the above laboratory data obtained from a single blood draw. For the analysis of FCPR values under the assay detection limit of 0.001 nmol/l, an approximate value of 0.001 nmol/l was used. Correlations between SD and glycaemic variability indices were also tested using multivariate analyses. To obtain the cut-off values for GA/A1c and FCPR index that best discriminated between patients with lower and higher SDs, we repeated receiver operating characteristic (ROC) analysis using serial cut-off values until we found the cut-off value that would create the highest AUC. The disparity of SD, as well as the sensitivity, specificity, and AUC, were calculated after dividing the patients into two groups.

## Results

**Figure 1:**
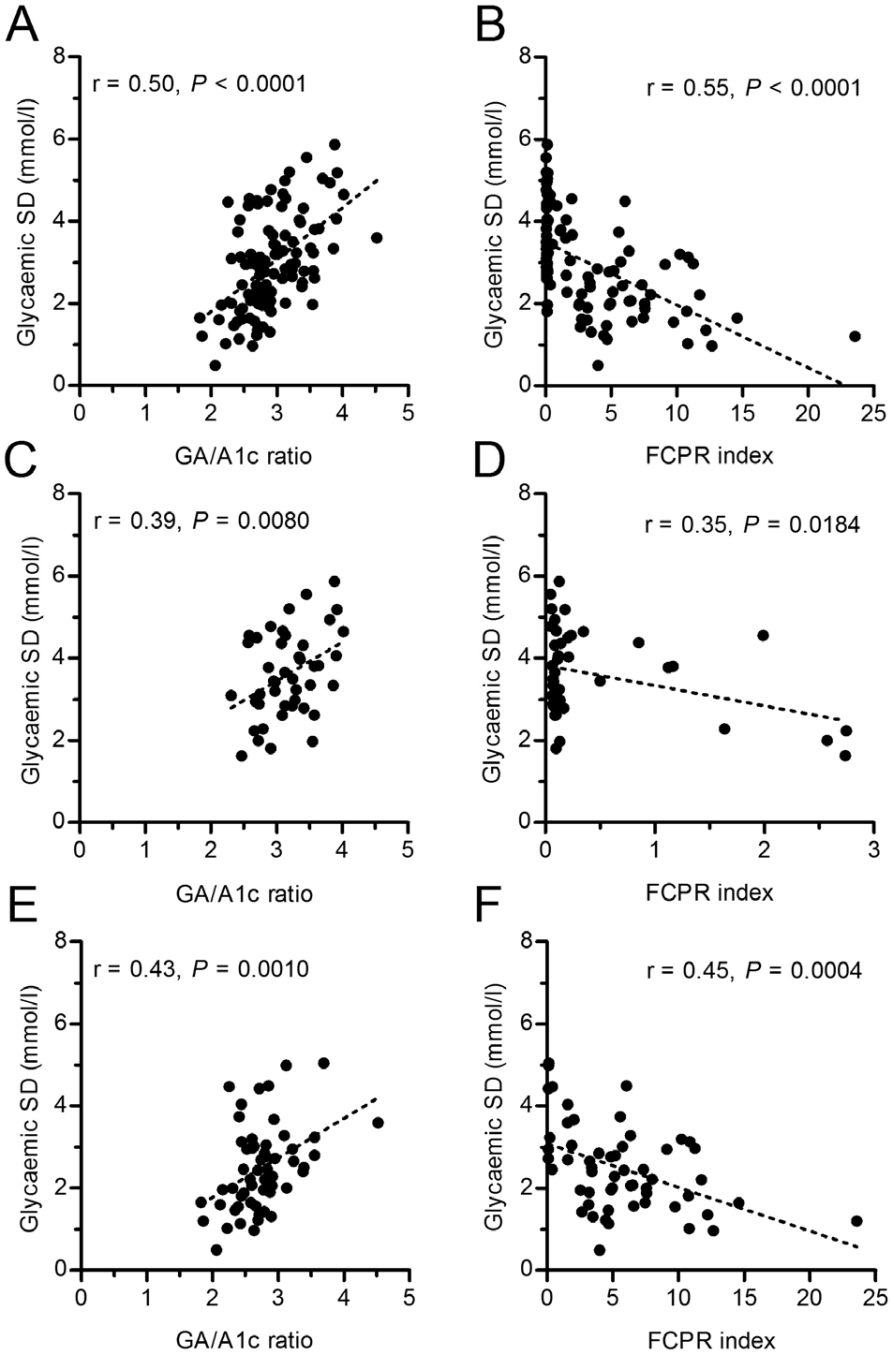
Association between glycaemic variability and its predictive indices. Standard deviation (SD) of serial glycaemic values obtained with 48-h CGM recordings were analyzed against GA/A1c ratio (A) and FCPR index (FCPR/FPG×100) (B) using Pearson's univariate correlation analysis in 100 diabetic patients. The analyses were carried out separately in 44 type 1 (C, D) and 56 type 2 (E, F) diabetic patients. Dotted lines represent regression lines and *r* represents correlation coefficient.

**Figure 2:**
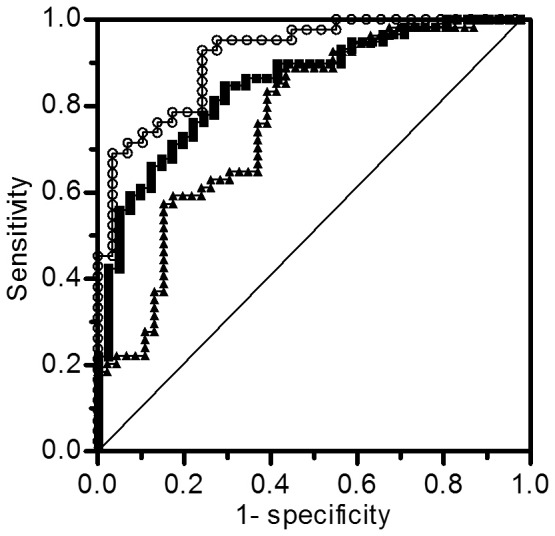
Receiver operating characteristic (ROC) curves for GA/A1c ratio and FCPR index (FCPR/FPG×100) for predicting glycaemic variability indices. ROC analyses were repeated using serial cut-off data for GA/A1c ratio and FCPR index, and the greatest AUC of 0.7665 for GA/A1c ratio (closed triangles) and of 0.8504 for FCPR index (closed squares) was obtained when the cut-off values were set at 2.8 and 3.0, respectively. ROC analysis simultaneously using a GA/A1c ratio of 2.8 and FCPR index of 3.0 to predict glycaemic fluctuations yielded an AUC of 0.9154 (open circles).

**Figure 3:**
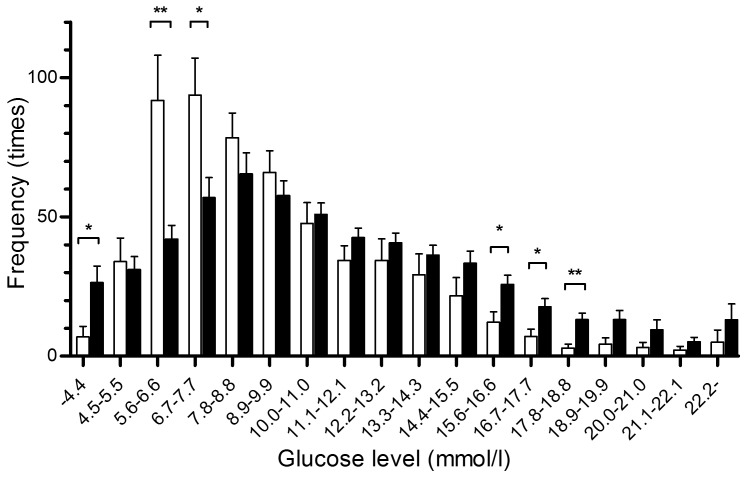
Distribution of glycaemic levels during 48-h CGM recording in two groups of diabetic patients predicted as having high and low glycaemic variabilities. Subcutaneous glycaemic data automatically recorded every 10 seconds and averaged every 5 min during 48-h CGM in 100 diabetic patients were stratified according to each glucose level, and the frequency showing each glucose range was plotted. Closed bars represent mean ± SEM of the frequency in diabetic patients with GA/A1c ratio ≥2.8 and FCPR index (FCPR/FPG×100) <3.0; open bars represent patients with GA/A1c ratio <2.8 and FCPR index ≥3.0. *P<0.05, **P<0.01.

Glycaemic SDs obtained from all CGM data in 100 diabetic patients showed a normal distribution on the D'Agostino-Pearson (omnibus K2) normality test with an average value of 3.0 mmol/l (K2 = 3.757, *P* = 0.1529, α = 0.05). Pearson's univariate correlation analysis revealed that SD was significantly associated with the type of diabetes and with body mass index (Table 2). SD was not associated with A1c, but showed a significant positive correlation with FPG, GA and GA/A1c ratio, and a negative correlation with FCPR and FCPR index (Table 2). Stepwise multiple linear regression analysis of these items revealed that only GA/A1c ratio (Fig. 1A) and FCPR index (Fig. 1B) were significantly correlated with glycaemic SD. Such significant correlations did not disappear when the type 1 (Fig. 1C, 1D) and type 2 (Fig. 1E, 1F) diabetes groups were analyzed separately. The type of diabetes was not independently related to glycaemic SD. GA/A1c ratio and FCPR index showed negative and positive correlations, respectively, with the nine other previously reported glucose variability formulas (Table 3). To obtain a cut-off value that best discriminated between patients with smaller and larger SD values, ROC curves were plotted using serial cut-off data for GA/A1c ratio and FCPR index. ROC analysis revealed the greatest AUC of 0.7665 (95% confidence interval (CI) 0.6726–0.8604, *P*<0.0001) when the cut-off value for GA/A1c ratio was set at 2.8, with a sensitivity of 83%, a specificity of 61% and a SD value of 2.5 mmol/l (Fig. 2). The group with GA/A1c ratios ≥2.8 showed distinctly larger SDs than those with GA/A1c ratios <2.8 (3.5±1.0 mmol/l *vs.* 2.4±1.1 mmol/l, *P*<0.0001). The AUC for FCPR index was greatest at 0.8504 (95% CI 0.7761–0.9246, *P*<0.0001) when a cut-off value of 3.0 was set, with a SD of 2.6 mmol/l, a sensitivity of 85%, and a specificity of 71% (Fig. 2). Patients with FCPR index <3.0 showed distinctly larger SDs than those with FCPR index ≥3.0 (3.5±1.0 mmol/l *vs.* 2.2±0.8 mmol/l, *P*<0.0001). ROC analysis demonstrated that patients showing both GA/A1c ratio ≥2.8 and FCPR index <3.0 had distinctly higher glycaemic variability than those with GA/A1c ratio <2.8 and FCPR index ≥3.0 (mean ± SD of glycaemic SD values: 3.7±1.0 mmol/l *vs.* 2.0±0.8 mmol/l, respectively). The AUC was 0.9154 (95% CI 0.8523–0.9786, *P*<0.0001) (Fig. 2). The results indicate the validity of GA/A1c ratio ≥2.8 and FCPR index <3.0 as predictors of high glycaemic variability. The clinical relevance of the combination of GA/A1c ratio ≥2.8 and FCPR index <3.0 to glycaemic variability was investigated. The A1c values in patients with these values were not significantly different from those in patients with a GA/A1c ratio <2.8 and FCPR index ≥3.0 (72±22 *vs.* 73±18 mmol/mol [IFCC], 8.8±2.1 *vs.* 8.8±1.6% [NGSP], n.s.). Patients showing a GA/A1c ratio ≥2.8 and FCPR index <3.0 (n = 42) experienced significantly longer durations of hypoglycaemia under 4.4 mmol/l and hyperglycaemia between 15.6–18.8 mmol/l, compared with those with a lower GA/A1c ratio and higher FCPR index value (n = 30) (Fig. 3). This suggests that a GA/A1c ratio ≥2.8 plus FCPR index <3.0 could serve as an indicator for greater glycaemic variability and might thus predict more frequent hypoglycaemic and hyperglycaemic episodes. Patients not meeting these criteria were in the normoglycaemic range (5.6–7.7 mmol/l) for significantly longer periods of time and showed less hypoglycaemia (Fig. 3). Finally, we analysed the ability of the above indices to predict glycaemic variability even when patients with undetectable CPR levels and those with detectable CPR levels were analysed separately, irrespective of the type of diabetes. In 68 patients with detectable CPR levels (16 type 1 and 52 type 2 patients), glycaemic SD again closely correlated with both GA/A1c ratio (r = 0.36, *P* = 0.0025) and FCPR index (r = −0.45, *P* = 0.0001). GA/A1c ratio also correlated significantly with glycaemic SD in the remaining 32 patients with undetectable CPR levels (r = 0.37, *P* = 0.0318).

**Table 2:** Univariate and multivariate correlation analysis of 100 patients.

		Univariate		Multivariate			
Factors	Parameters	r	*P*	R^2^	β	F	*P*
Glycaemic SD	Type 1/2	−0.48	<0.0001	0.40	0.18	3.00	0.0861
(mmol/l)	Sex M/F	−0.01	0.9875				
	Age (years)	−0.02	0.7868				
	BMI (kg/m^2^)	−0.27	0.0058				
	Duration of diabetes (years)	0.18	0.0791				
	FPG (mmol/l)	0.21	0.0351				
	HbA_1c_ (mmol/mol, IFCC)	−0.05	0.6030				
	HbA_1c_ (%, NGSP)	−0.05	0.6030				
	GA (%)	0.31	0.0016				
	FCPR (nmol/l)	−0.51	<0.0001				
	GA/A1c ratio	0.53	<0.0001	0.38	0.33	12.16	0.0007
	FCPR index	−0.55	<0.0001	0.30	−0.37	14.65	<0.0001

Univariate, linear regression analysis; Multivariate, stepwised multivariate analysis.

BMI, body mass index; FPG, fasting plasma glucose; HbA_1c_, glycated haemoglobin; GA, glycated albumin; FCPR, fasting serum C-peptide immnoreactivity; GA/A1c ratio, glycated albumin to glycated haemoglobin ratio; FCPR index, FCPR to FPG ratio (FCPR/FPG×100).

**Table 3:** Association of GA/A1c ratio and FCPR index with 9 other glucose variability formulas of 100 patients.

	GA/A1c ratio		FCPR index	
Parameters	r	*P*	r	*P*
%CV (%)	0.35	0.0003	−0.45	<0.0001
Range (mmol/l)	0.47	<0.0001	−0.54	<0.0001
IQR	0.45	<0.0001	−0.49	<0.0001
J-index	0.41	<0.0001	−0.35	0.0003
MAGE	0.46	0.0001	−0.43	<0.0001
M_5.6_	0.34	0.0006	−0.29	0.0039
IGC	0.34	0.0006	−0.37	0.0002
MODD	0.40	<0.0001	−0.52	<0.0001
CONGA_1–24_	0.35	0.0004	−0.41	<0.0001

GA/A1c ratio, GA/HbA1c ratio; FCPR index, fasting plasma C-peptide/fasting plasma glucose×100; %CV, % coefficient of variation; Range, the difference between the highest and lowest interstitial glucose levels; IQR, Interquartile range; J-index, 0.001×(mean glucose value+SD)^2^; MAGE, mean amplitude of glycemic excursions; M_5.6_, M_R_ with R = 5.6 mmol/l (100 mg/dl); IGC, index of glycemic control; MODD, mean of daily difference; CONGA, continuous overlapping net glycemic action.

## Discussion

The present study confirmed the results of a previous report suggesting that glycaemic SD is correlated with GA but not with A1c [11] and further showed that glycaemic SD is more strongly associated with the GA/A1c ratio than with a single GA value alone. The current study found a remarkable difference in glycaemic SD values between patients with and without GA/A1c ratio ≥2.8, even though there was no significant difference in A1c values. A GA/A1c ratio of 2.8 could thus serve as a cut-off value for discriminating between patients with smaller and larger SD values. However, because the half-life of GA is far shorter than that of A1c, the GA/A1c ratio may theoretically increase in the event of recently deteriorating glycaemic control, independently of glycaemic fluctuations [24]. We were therefore fortunate to identify additional indices predictive of glycaemic variability, which could be obtained from a single fasting blood test. FCPR had a significant negative correlation with SD, while FCPR index showed a stronger negative correlation with glycaemic SD, with no significant correlation with average blood glucose or A1c. Because the cut-off level for FCPR for differentiating between patients likely to show high and low glycaemic variability was very low (<0.2 nmol/l), using FCPR index allowed efficient discrimination between the two groups. Overall, the results indicate that basal β-cell dysfunction may result in a larger glycaemic fluctuation, and that a FCPR index value of 3.0 may serve to discriminate between patients with high and low glycaemic fluctuations.

Despite the close correlation between basal β-cell function and glycaemic SD, in addition to the discriminative power of an FCPR index value of 3.0 for patients with high and low glycaemic variability, the question of whether patients initially showing low FCPR values whose glycaemic control and fluctuations have been markedly improved and stabilized by proper insulin and dietary therapy may still show low values of FCPR index remains. The FCPR index is heavily dependent upon the size of the FCPR in the numerator, even if FPG approaches a normal value. In the current study, however, most type 2 patients with reduced FCPR levels and lower glycaemic SD after successful treatment with insulin showed some recovery of β cell function and reduced FPG. Therefore, many such patients could be grouped in the low SD category based on FCPR index. However, GA/A1c ratio and FCPR index used simultaneously identified patients at high risk of glycaemic variation with high sensitivity and specificity, compared with the use of a single index.

We also investigated disparities in the blood glucose profiles of the two groups classified as having the highest and lowest glycaemic variabilities, by using both indices. The patient group with the highest glycaemic SD showed blood glucose levels exceeding 10 mmol/l during longer periods of time than the group with the lowest SD. Notably, the former group experienced markedly longer hypoglycaemic periods and far shorter normoglycaemic periods compared with the latter group. Thus, patients presenting GA/A1c ratios ≥2.8 and FCPR indices <3.0 are expected to show high glycaemic variability and to be at greater risk of hypoglycaemia, despite their A1c levels.

It should be emphasised that glycaemic SD was closely correlated with GA/A1c ratio and FCPR index, even when type 1 and type 2 diabetes were analysed separately, and when patients with detectable and undetectable FCPR levels were tested separately. Glycaemic SD also correlated with GA/A1c ratio and FCPR index when insulin-treated patients and oral-reagent-alone patients were analysed separately (data not shown). These results demonstrate the clinical usefulness of the indices as potential predictors of high glycaemic variability, irrespective of diabetes type or basal β-cell function.

In conclusion, high GA/A1c ratio and low FCPR index are associated with higher glycaemic variability. Simultaneous measurements of GA, A1c, FPG and FCPR could potentially identify patients likely to have high glycaemic variability and/or hypoglycaemia.
